# Differential activation of p38 and extracellular signal-regulated kinase in spinal cord in a model of bee venom-induced inflammation and hyperalgesia

**DOI:** 10.1186/1744-8069-4-17

**Published:** 2008-04-30

**Authors:** Xiu-Yu Cui, Yi Dai, Sheng-Lan Wang, Hiroki Yamanaka, Kimiko Kobayashi, Koichi Obata, Jun Chen, Koichi Noguchi

**Affiliations:** 1 Department of Anatomy and Neuroscience, Hyogo College of Medicine, Nishinomiya, Hyogo 663-8501, Japan; 2 Institute for Biomedical Sciences of Pain, Capital Medical University, Beijing 100069, PR China; 3 Department of pharmacy, School of pharmacy, Hyogo University of Health Sciences, Kobe, Hyogo 650-8530, Japan; 4 Institute for Biomedical Sciences of Pain and Institute for Functional Brain Disorder Tangdu Hospital, Fourth Military Medical University, Xi'an 710038, PR China

## Abstract

**Background:**

Honeybee's sting on human skin can induce ongoing pain, hyperalgesia and inflammation. Injection of bee venom (BV) into the intraplantar surface of the rat hindpaw induces an early onset of spontaneous pain followed by a lasting thermal and mechanical hypersensitivity in the affected paw. The underlying mechanisms of BV-induced thermal and mechanical hypersensitivity are, however, poorly understood. In the present study, we investigated the role of mitogen-activated protein kinase (MAPK) in the generation of BV-induced pain hypersensitivity.

**Results:**

We found that BV injection resulted in a quick activation of p38, predominantly in the L4/L5 spinal dorsal horn ipsilateral to the inflammation from 1 hr to 7 d post-injection. Phosphorylated p38 (p-p38) was expressed in both neurons and microglia, but not in astrocytes. Intrathecal administration of the p38 inhibitor, SB203580, prevented BV-induced thermal hypersensitivity from 1 hr to 3 d, but had no effect on mechanical hypersensitivity. Activated ERK1/2 was observed exclusively in neurons in the L4/L5 dorsal horn from 2 min to 1 d, peaking at 2 min after BV injection. Intrathecal administration of the MEK inhibitor, U0126, prevented both mechanical and thermal hypersensitivity from 1 hr to 2 d. p-ERK1/2 and p-p38 were expressed in neurons in distinct regions of the L4/L5 dorsal horn; p-ERK1/2 was mainly in lamina I, while p-p38 was mainly in lamina II of the dorsal horn.

**Conclusion:**

The results indicate that differential activation of p38 and ERK1/2 in the dorsal horn may contribute to the generation and development of BV-induced pain hypersensitivity by different mechanisms.

## Background

Honeybee's sting on human skin can induce ongoing pain, hyperalgesia and inflammation. Intraplantar injection (i.pl.) of bee venom (BV) as an inflammatory pain model has been widely used [1-3]. Our previous behavioral studies have demonstrated that i.pl. of BV in awake rats could produce a persistent or tonic spontaneous nociception, followed by long-term thermal and mechanical hyperalgesia, and peripheral inflammation [2,4,5]. BV-induced peripheral inflammatory symptoms include the skin becoming red, swollen, hot and aching which are totally in accordance with the clinical inflammatory symptoms. Our previous electrophysiological experiments suggest that the BV model possesses many advantages over the formalin test, another inflammatory pain model, and may be more appropriate to use in the evaluation of the mechanisms underlying clinical pathological pain [2,6-8].

The mitogen-activated protein kinases (MAPKs) are a family of serine/threonine protein kinases, which exist in a variety of cells. They transduce a broad range of extracellular stimuli into diverse intracellular responses by producing changes in transcriptional modulations of key genes, as well as posttranslational modifications of target proteins [9,10]. There are four main MAPKs family members in mammalian cells: extracellular signal-regulated kinase1/2 (ERK1/2), p38, c-Jun N-terminal kinase (JNK), and ERK5, which contribute to different signal transduction systems [11,12]. Within the past decade, several studies in rodents have elucidated the roles of ERK, p38, JNK and ERK5 in generating nociceptive sensitivity and nociceptive plasticity. The activation and the role of MAPKs in nociceptive plasticity have been extensively studied in the spinal cord and dorsal root ganglia (DRG). ERK1/2 is activated during noxious, but not innocuous stimulation [13,14]. ERK1/2 activation is found in the spinal cord dorsal horn under inflammatory pain conditions induced by complete Freund's adjuvant (CFA) [14], mustard oil [15], formalin [16,17], or carrageenan [18]. It is believed that ERK1/2 activation in the spinal cord dorsal horn is involved in spinal nociceptive processing, neuronal plasticity and central sensitization under inflammatory pain conditions [12,14,16,19]. p38 can be activated in the spinal cord dorsal horn by intraplantar administration of formalin [20,21] or capsaicin [22]. bActivated p38 in the spinal cord is thought to play an important role in inflammation-induced spinal hyperalgesia [21,23].

It is not clear whether i.pl BV injection induces activation of MAPK family members in neurons or glial cells in the spinal cord, and whether their activation contributes to BV-induced persistent thermal or mechanical hypersensitivity. In the present study, using immunohistochemistry and behavioral test, we investigated the expression of activated MAPKs in detail in the spinal cord after i.pl. BV injection. Further, the functional role of differential activation of MAPKs in BV-induced peripheral inflammatory pain in different cells are reported and discussed.

## Results

### p38 activation in the spinal cord in the BV-inflamed rats

**Figure 1:**
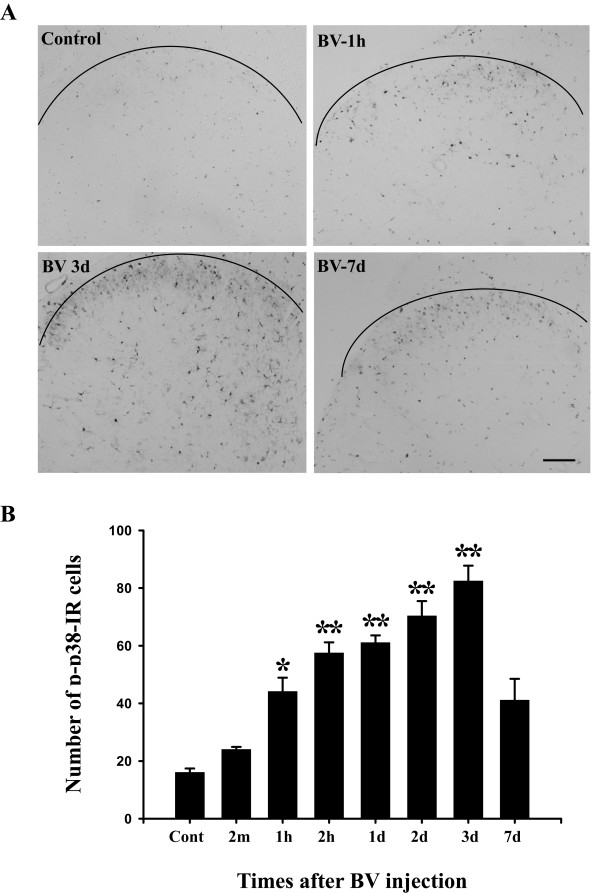
BV injection evoked p38 phosphorylation in rat dorsal horn. (A) p-p38 immunostaining in an L4/5 spinal cord section (16 μm) in a control and at the indicated time points after intraplantar BV injection (200 μg/50 μl). Immunohistochemistry shows a gradual increase in the number of p-p38 immunoreactive cells in the ipsilateral spinal cord. (B) Time course of BV-evoked p-p38 labeled cells in the lamina I-II of L4/5 dorsal horn (n = 4 or 5 each time point, ** p < 0.001; *p < 0.05; compared with control; one-way ANOVA). Scale bars, 100 μm; cont, control.

p-p38 immunohistochemistry showed a low constitutive expression in the L4/5 spinal dorsal horn in naive group or after saline injection (Fig. 1A, control). The number of p-p38 labeled cells was slightly increased at 2 min after BV injection. The number and intensity of p-p38-IR cells began to increase more obviously and significantly at 1 hr and was further increased at 2 hr and 1 d. Three days after BV injection, the increase in the number and intensity of p-p38-IR cells peaked in the ipsilateral L4/L5 spinal cord (Fig. 1A,B). The most prominent increase was found in laminae I-II of the dorsal horn, but the deep dorsal horn (laminae III-V) also showed an increase in p-p38-IR cells (Fig. 1A, 3d).

The total number of p-p38-IR cells in laminae I-II of the spinal cord was 16.1 ± 1.4 in the control group and 24.0 ± 0.9 in the BV group 2 min after injection. There was no significant difference between the control group and the BV 2 min group (p = 0.960). The total number of p-p38-IR cells was significantly increased at 1 hr, 2 hr, 1 d, 2 d after BV injection and reached a peak at 3 d (Fig. 1B). Then the number of p-p38-IR cells decreased at 7 d to a level that was not significantly different from the control group. These data showed that BV injection significantly induced p38 activation from 1 hr to 3 d, at which point p38 activation began to decrease.

**Figure 2:**
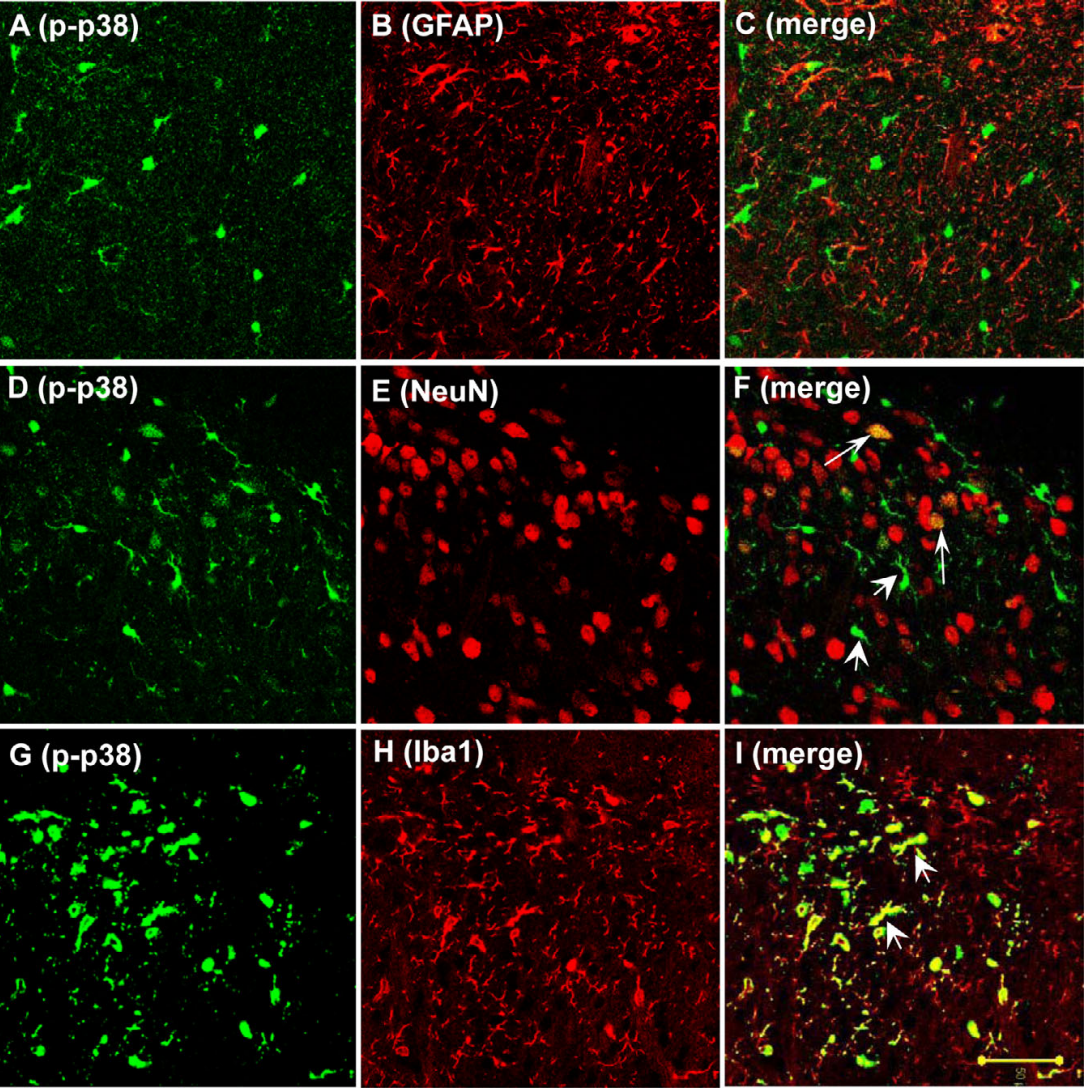
BV induced p38 phosphorylation in both neurons and microglia in the rat L4/5 spinal dorsal horn. (A-I) Double immunofluorescence in the dorsal horn of L4/5 spinal cord sections (16 μm) at 3 d after BV-injection, for p-p38 (green) and GFAP, an astrocyte marker (red; B, C); NeuN, a neuronal marker (red; E, F); and Iba1, a microglia marker (red; H, I) (40×). (A-C) p-p38-IR was not co-expressed with GFAP-IR. (D-F) Double immunofluorescence for p-p38 and NeuN indicated partial colocalization of p-p38 and NeuN (F, white arrow). There were many p-p38-IR cells that are not co-expressed with NeuN in the dorsal horn after BV injection (F, white arrow head). (G-I) p-p38-IR which is not co-expressed with NeuN colocalized with Iba1 (I, white arrow head). Scale bars, 50 μm (A-I).

In order to identify the cell types which expressed p-p38 in the dorsal horn after BV injection, we performed double immunostaining of p-p38 with several cell-specific markers: NeuN (neuron); GFAP (astrocyte); and Iba1 (microglia). p-p38 immunoreactive cells did not co-express GFAP (Fig. 2A–C), indicating that p-p38 positive cells were not astrocytes. The cell types labeled with p-p38 labeled varied; p-p38-IR was partially co-expressed with NeuN (Fig. 2D–F) and also co-expressed with Iba1 (Fig. 2G–I). The results indicated that p38 was activated in both neurons and microglia under BV-induced peripheral inflammation.

**Figure 3:**
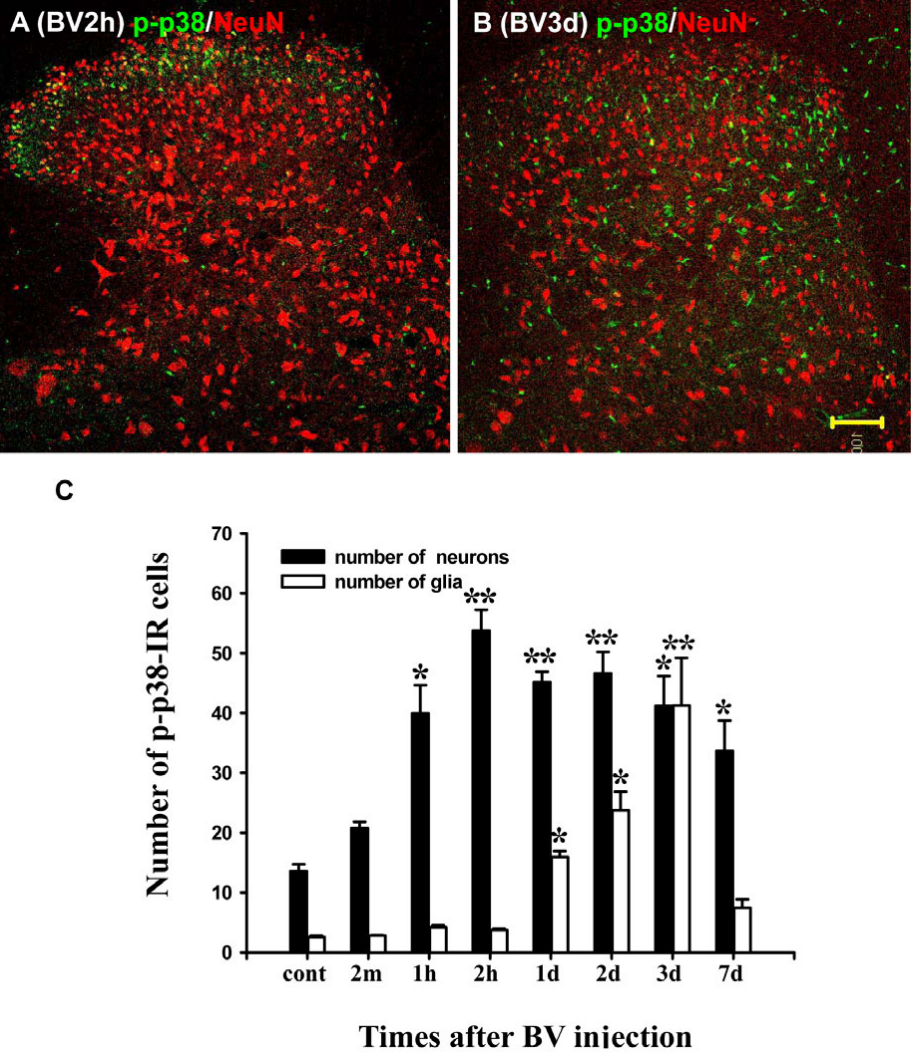
BV induced different activation patterns of p38 in neurons and microglia in the L4/5 rat spinal dorsal horn. (A-B) Double immunofluorescence in the dorsal horn of L4/5 spinal cord sections (16 μm) for p-p38 (green) and NeuN, a neuronal marker (red) at 2 h and 4 d after BV injection (10×). (C) Time course showing the number of p-p38-IR neurons and microglia in the I-II laminae in L4/5 dorsal horn. Double immunofluorescence and histograms show that p38 was activated in neurons as early as 1 hr and peaked at 2 hr; while p38 was activated in microglia at 1 d and peaked at 3 d after BV administration. (n = 4 or 5 each time point, ** p < 0.001; *p < 0.05; compared with controls; one-way AVOVA). Scale bars, 100 μm (A, B). cont, control.

BV administration induced a differential activation pattern of p38 in neurons and microglia. The p-p38 labeled cells mainly co-expressed NeuN at 1 hr or 2 hr after BV injection (Fig. 3A). The p-p38-IR cells that co-expressed Iba1, not NeuN, began to increase at 1 d and remained at a high level until 3 d after BV injection (Fig. 3B). However, at 7 d the majority p-p38-IR cells were NeuN-IR (data not shown) and the p-p38 labeled microglia returned to control level. We counted the number of p-p38-IR neurons and the number of p-p38-IR microglia in lamina I-II of the dorsal horn (Fig. 3C). Few microglia expressed p-p38-IR in the control dorsal horn (2.6 ± 0.3) (Fig. 3C), and the number of p-p38-IR microglia in the dorsal horn did not increase significantly from 2 min to 2 hr after BV injection compared with that of the controls. In contrast, the number of p-p38-IR neurons increased significantly compared with the controls from 1 hr to 7 d after BV injection and it peaked at 2 hr (Fig. 3C). The number of p-p38-IR microglia increased significantly from 1 d to 3 d after BV injection, and it peaked at 3 d, then decreased to the control level at 7 d (Fig. 3C).

### ERK1/2 activation in the spinal cord in BV-inflamed rats

**Figure 4:**
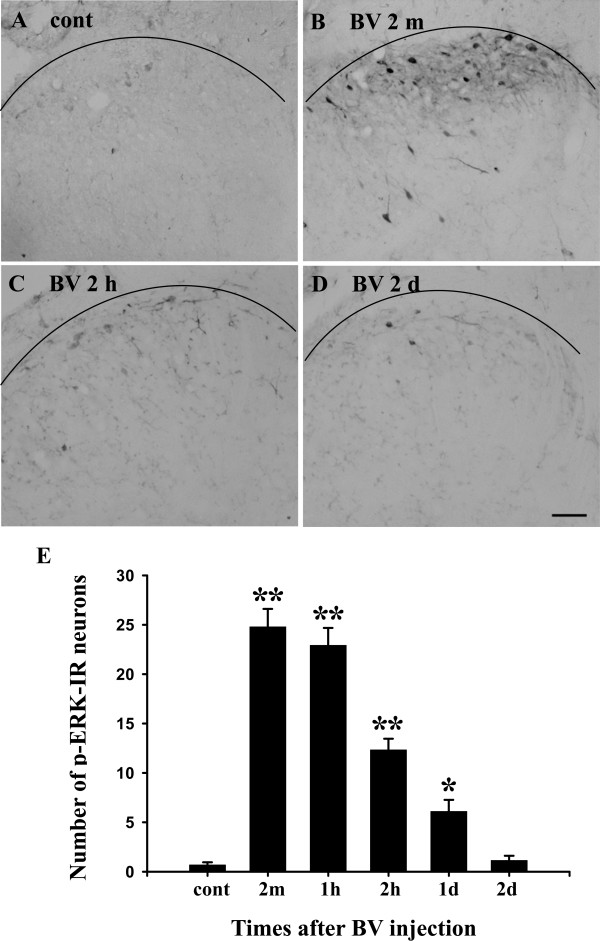
ERK was phosphorylated in the rat L4/5 spinal dorsal horn after BV injection. (A) Control p-ERK immunostaining in an L4/5 spinal cord section (16 μm) after intraplantar saline injection (50 μl). Only a few of p-ERK-IR cells were detected. (B-D) p-ERK immunostaining in L4/5 spinal cord sections (16 μm) at 2 min, 2 hr, and 2 d after intraplantar BV injection (200 μg/50 μl). p-ERK-IR cells were distributed mainly in laminae I-II of the spinal dorsal horn. Immunohistochemistry indicates a rapid increase in the number of p-ERK immunoreactive cells in the ipsilateral spinal cord at 2 min. (E) Time course of BV-evoked p-ERK labeled cells in the L4/5 dorsal horn. (n = 4 or 5 each time point, ** p < 0.001; *p < 0.05; compared with controls; one-way AVOVA). Scale bars, 50 μm in A-D. cont, control.

We next examined whether BV-induced persistent peripheral inflammation also induced ERK1/2 activation in the spinal cord dorsal horn. Few cells expressed p-ERK1/2 in the spinal dorsal horn of naive or saline-treated rats (Fig. 4A). BV administration induced ERK1/2 activation in the spinal dorsal horn as early as 2 min after BV injection (Fig. 4B). Activated ERK1/2 was found in the nucleus, cytoplasm and dendrites of dorsal horn neurons. The significant increase in the number of p-ERK1/2-IR cells was observed primarily in the superficial dorsal horn ipsilateral to the side of BV injection (Fig. 4B–D). ERK1/2 activation was not found on the contralateral side (data not shown). The number of p-ERK1/2-IR cells peaked at 2 min, remained at a high level at 1 hr and decreased over the next 24 hr after BV injection (Fig. 4B–E). We counted the number of p-ERK1/2-IR cells in the laminae I-II of the dorsal horn in control, 2 min, 1 hr, 2 hr, 1 d, and 2 d after BV injection (Fig. 4E). The number of p-ERK1/2-IR cells was 0.68 ± 0.3 in controls, and the number of p-ERK1/2-IR cells significantly increased at 2 min, 1 hr, 2 hr and 1 d and returned to the control level at 2 d (Fig. 4E).

**Figure 5:**
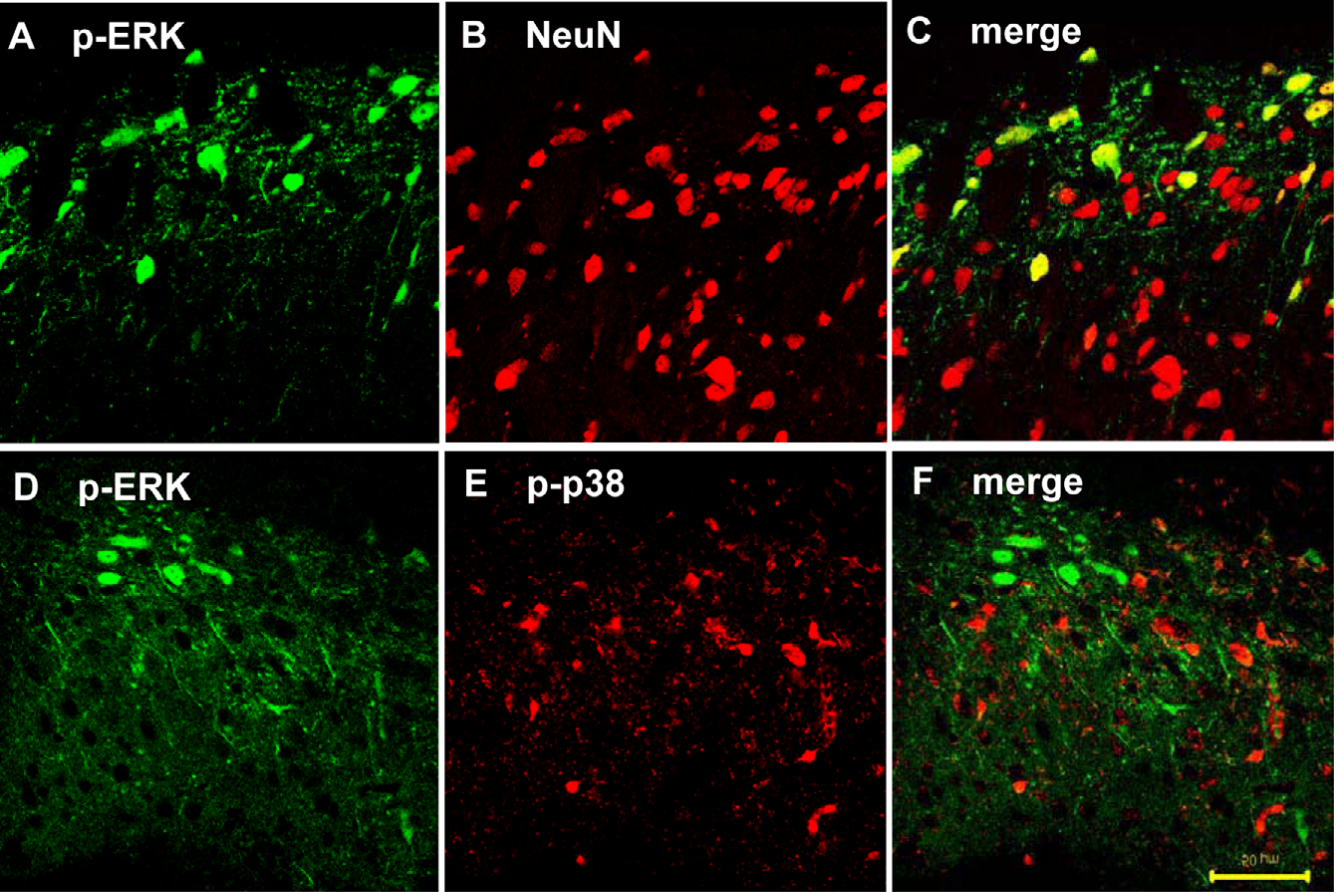
BV induced ERK phosphorylation only in neurons that did not co-express p-p38 in the rat L4/5 spinal dorsal horn. (A-C) Double immunofluorescence in spinal dorsal horn sections (16 μm) for p-ERK (green) and NeuN, a neuronal marker (red). Double immunofluorescence indicated significant colocalization between p-ERK and NeuN. (D-F) Double immunofluorescence in the dorsal horn of an L4/L5 spinal cord section (16 μm) for p-ERK (green) and p-p38 (red). Double immunofluorescence indicated p-ERK and p-p38 did not colocalize. Scale bars, 50 μm (A-F).

In order to identify the cell types that expressed p-ERK1/2 in the dorsal horn after BV injection, we performed double immunostaining of p-ERK1/2 with cell-specific markers. The p-ERK1/2 expressing cells did not express GFAP or Iba1 (data not shown), but all co-expressed NeuN (Fig. 5A–C). We also performed double immunostaining of p-ERK1/2 with p-p38 to determine whether both MAPKs were co-expressed after BV injection. The majority of p-ERK1/2-IR cells were in lamina I, however p-p38 labeled cells were mainly in lamina II of the spinal dorsal horn. p-ERK1/2-IR cells did not co-express p-p38-IR (Fig. 5D–F).

### Intrathecal administration of a p38 inhibitor, SB203580, inhibited BV-induced thermal hypersensitivity, but not mechanical hypersensitivity

**Figure 6:**
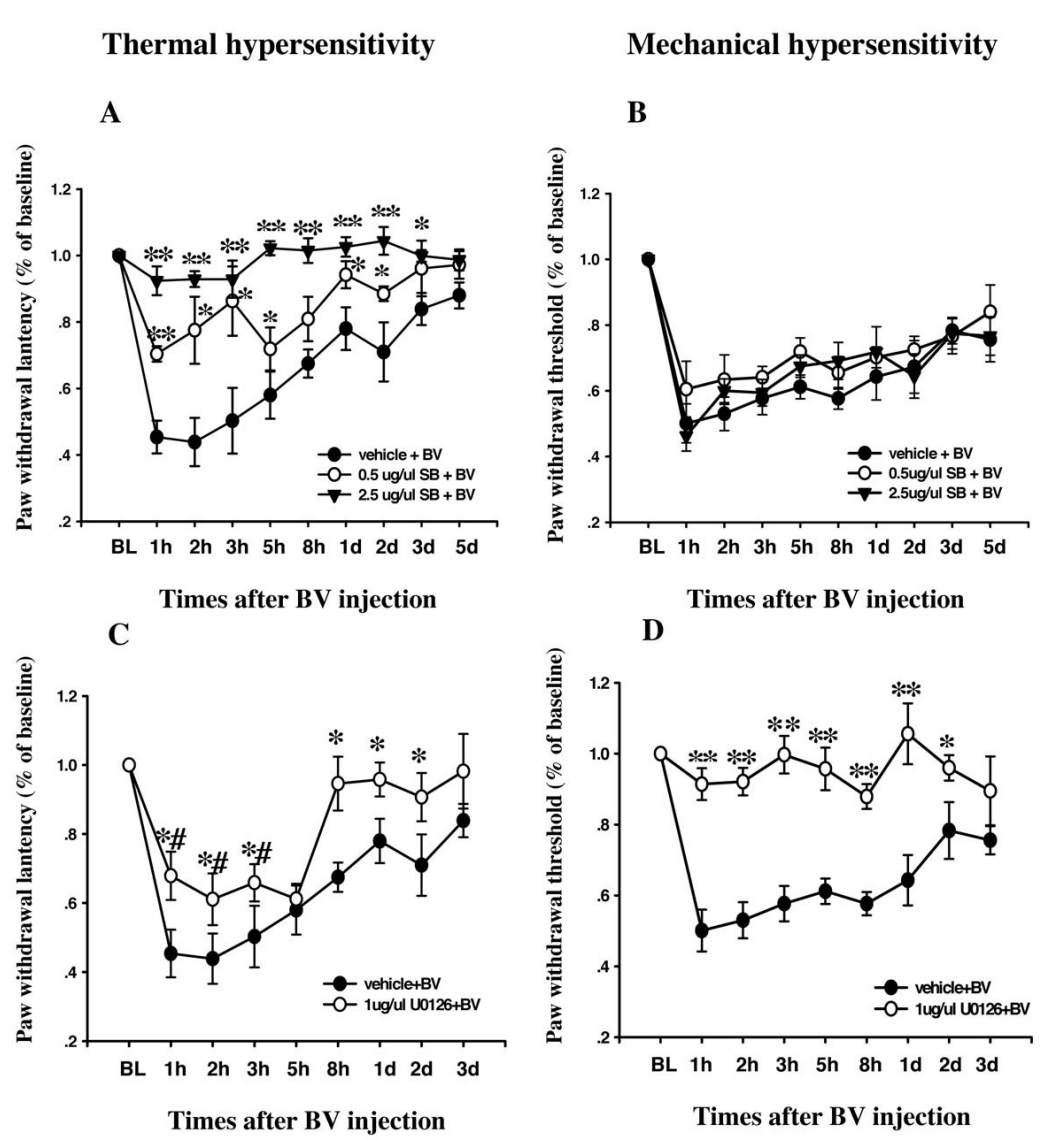
Effects of pre-administration of SB203580 or U0126 on the induction and maintenance of BV-induced thermal and mechanical hypersensitivity. The paw withdrawal latencies or thresholds at different time points after BV injection are presented as the ratio compared with the baseline. (n = 6 or 5 each time point, ** p < 0.001; *p < 0.05; compared with vehicle; t-test #: p < 0.05; compared with baseline, AVOVA). BL, baseline.

BV injection into the plantar surface of the hindpaw in the rat induced both mechanical and thermal hypersensitivity in the hindpaw from 1 hr to 3 d after BV injection [2], while in the present study p38 was activated in the dorsal horn from 1 hr to 7 d after BV injection. To investigate whether p38 activation has an effect on the development of mechanical and thermal hypersensitivity after BV injection, we continuously administered vehicle (10% DMSO) or SB203580, a specific p38 inhibitor, into the intrathecal space with a mini-osmotic pump 12 hr before BV injection and lasting for 3 d. We compared paw withdrawal latency at different time points after BV injection to the baseline that was measured before BV injection. Vehicle treatment had no effect on BV-induced peripheral thermal and mechanical hypersensitivity (Fig. 6A,B). Intrathecal administration of SB203580 dose-dependently prevented BV-induced thermal hypersensitivity. Intrathecal administration of 0.5 μg/μl SB 203580 significantly but partially prolonged paw withdrawal latency from 1 hr to 2 d after BV injection. A higher dose of SB 203580, 2.5 μg/μl, significantly prolonged paw withdrawal latency from 1 hr to 3 d after BV injection (Fig. 6A). The results indicated that intrathecal administration of 2.5 μg/μl SB 203580 completely prevented the thermal hypersensitivity induced by BV injection.

In contrast, intrathecal administration of 0.5 μg/μl or 2.5 μg/μl SB 203580 had no significant effects on mechanical hypersensitivity after BV injection. The paw withdrawal threshold was not significant different between the 0.5 μg/μl SB 203580, 2.5 μg/μl SB203580 and vehicle groups (Fig. 6B).

### Intrathecal administration of the MEK inhibitor, U0126, inhibited both BV-induced thermal and mechanical hypersensitivity

To examine the functional role of ERK1/2 activation in BV-induced inflammatory pain, we continuously administered vehicle (10% DMSO) or 1 μg/μl U0126, a potent and selective MEK inhibitor, which was dissolved in 10% DMSO, into the intrathecal space with a mini-osmotic pump (1 μl per hour) 12 hr before BV injection and lasting for 3 d. Intrathecal U0126 and vehicle administration had no effect on basal thermal and mechanical behavior. The U0126 dose used was in accordance with previous work. Vehicle treatment had no obvious effect on BV-induced peripheral thermal and mechanical hypersensitivity (Fig. 6C,D).

Intrathecal administration of U0126 significantly, but not completely, prevented BV-induced thermal hypersensitivity. Intrathecal administration of 1 μg/μl U0126 significantly prevented the paw withdrawal latencies compared with vehicle level from 1 hr to 2 d after BV injection (Fig. 6C). For mechanical hyperalgesia, the intrathecal administration of 1 μg/μl U0126 significantly and completely prevented the mechanical hyperalgesia induced by BV injection (Fig. 6D). The paw withdrawal thresholds at different time points were not significantly different after U0126 treatment (Fig. 6D). The results indicated that intrathecal administration of 1 μg/μl U0126 completely prevented mechanical hyperalgesia, but only partly prevented thermal hypersensitivity induced by BV injection.

## Discussion

In this study, we investigated the activation and functional role of the MAPKs family (ERK1/2, p38) in the spinal cord in the BV-induced inflammatory pain model [2,24]. The present findings are comprised of four key observations: (1) BV injection induced persistent p38 activation in both spinal neurons and microglia. The activation of p38 in neurons occurred from 1 hr, while in microglia it started from 1 d after BV injection. (2) Intrathecal administration of the p38 inhibitor, SB203580, prevented thermal but not mechanical hyperalgesia induced by BV from 1 hr to 3 d. (3) BV injection induced ERK1/2 activation in spinal neurons from 2 min to 1 d, but not throughout the time course of activation observed in microglia. (4) Inhibition of ERK1/2 activation by the MEK inhibitor, U0126, prevented both thermal and mechanical hyperalgesia induced by BV from 1 hr to 2 d.

### BV induced p38-activation in the spinal dorsal horn

p38, a member of the MAPK family, is activated by cellular stress and inflammatory cytokines [11]. In the present study, we found that p38 was activated in both spinal neurons and microglia after BV injection into the plantar surface of the hindpaw. The number of p-p38 expressing cells was significantly increased from 1 hr to 7 d in the ipsilateral L4/5 spinal cord and peaked at 3 d (Fig 1). Intraplantar injection of BV induced tonic spontaneous nociceptive responses (flinching or licking and lifting of the injected paw) immediately and lasting for about 1 hr after injection and then was followed by long-term hyperalgesia. The number of p-p38-IR cells was not significantly increased at 2 min after BV injection, which indicates that p38 may not contribute to the onset of spontaneous pain. Our behavioral data showed BV-induced thermal and mechanical hyperalgesia was maintained from 1 hr to 3 d after BV-injection (Fig. 6, vehicle). The time courses of pain behavior and p-p38 expression coincided well with each other, suggesting a potential role of p38 activation in BV-induced pain hypersensitivity.

Interestingly, pretreatment with the p38 inhibitor dose-dependently inhibited the thermal hyperalgesia, but did not have any effect on the BV-induced mechanical hyperalgesia. These data suggest that p38 activation may play an important role in BV-induced thermal hyperalgesia, but not mechanical allodynia. Several lines of evidence have demonstrated that activation of p38 in the spinal cord is involved with the thermal hypersensitivity from peripheral inflammation induced by CFA, formalin or carrageenan [21,25]. p38 activation in the spinal cord is thought to be necessary for thermal hyperalgesia formation, therefore intrathecal administration of p-p38 inhibitors may inhibit the effect of activated p38 and the formation of thermal hyperalgesia [26,27]. In addition to the role of p38 in inflammatory pain, it has been reported that activation of p38 is induced by peripheral nerve injury. Administration of p38 inhibitors can block both thermal hyperalgesia [28,29] and mechanical allodynia [28,30,31] following peripheral nerve injury. Therefore, it seems likely that p-p38 is only involved in thermal hyperalgesia in inflammatory pain models, but is involved in both thermal and mechanical hyperalgesia in neuropathic pain models. Thus, p-p38 may play different roles under inflammatory and neuropathic pain conditions.

Double immunostaining of p-p38 with several cell-specific markers indicated that p-p38 was expressed in both neurons and microglia; the number of p-p38-IR neurons was significantly increased from 1 hr after BV injection and was maintained at a high level until 7 d. The number of p-p38-IR microglia was significantly increased from 1 d and peaked at 3 d after BV injection and then decreased to control level (Fig. 3). Our behavior data indicated that both thermal and mechanical hyperalgesia were induced from 1 hr and peaked within 3 d. However, activation of p38 in neurons continued for at least 7 d (Fig. 3). These data suggested that activation of p38 in neurons may be important to the induction, but not the maintenance, of BV-induced thermal hyperalgesia. Activation of p38 in microglia was induced from 1 d and peaked at 3 d, then returned to baseline by 7 d, which was completely consistent with the time course of thermal hyperalgesia. Thus, instead of a role in neurons, activation of p38 in microglia may contribute to the maintenance of BV-induced thermal hyperalgesia. It has been reported that p38 activation is induced in spinal microglia by CFA [21], carrageenan [25], or formalin intraplantar injection [19,32-34]. It is believed that p38 activation in microglia can worsen the inflammatory process by releasing proinflammatory mediators, which exert effects on neurons and contributes to pain hypersensitivity [35].

### BV-induced ERK1/2 activation in the spinal dorsal horn

Peripheral or central ERK pathways have been found to contribute to pain hypersensitivity in inflammatory and neuropathic pain models [13,14,16,36,37]. ERK activation in spinal dorsal horn neurons contributes to central sensitization through post-translational regulation processing at early times [14], and through transcriptional mechanisms at later times which leads to inflammatory pain hypersensitivity [19]. In the present study, we found that ERK1/2 was activated within 2 min in ipsilateral spinal neurons of lamina I-II, and maintained for as long as 24 hr after BV-injection. The rapid activation of ERK1/2 in the spinal cord may involve BV-induced tonic spontaneous nociceptive responses. In contrast to the expression of p-p38, p-ERK1/2 was induced exclusively in neurons. We found that p-ERK1/2 was not co-expressed with p-p38 in neurons in the dorsal horn after BV-injection. These data suggested that ERK1/2 and p38 were activated in separate cells following BV injection, and may contribute to neuronal hypersensitivity by different mechanisms. Indeed, pretreatment with the MEK inhibitor significantly prevented both thermal and mechanical hyperalgesia induced by BV injection, while the p38 inhibitor just inhibited the thermal hyperalgesia (Fig. 6).

ERK1/2 activation was found in the spinal cord following formalin, carrageenan or CFA intraplantar injection [14,18,19,38]. Formalin- or carrageenan-induced ERK activation is rapid and can be maintained for as long as 60 min [14,18], while CFA-induced ERK activation can persist in the spinal dorsal horn for about 48 hr [19], or even as long as 7 d [38]. We found that ERK1/2 was activated from 2 min and maintained as long as 24 hr in the ipsilateral spinal dorsal horn after BV injection. The temporal pattern in our results differs from the above-mentioned studies and this difference may be due to the differences in the employed animal models.

In the present study, using immunohistochemistry and behavioral tests, we investigated the expression and functional role of activated p38 or ERK1/2 in the spinal cord after i.pl. BV. Our data show that intraplantar BV-injection can cause activation of ERK1/2 only in neurons in the spinal dorsal horn, and cause activation of p38 in both spinal neurons and microglia. Intrathecal administration of a MEK inhibitor significantly prevented the BV-induced thermal and mechanical hyperalgesia, while the p38 inhibitor prevented thermal hyperalgesia but had no effect on mechanical hyperalgesia. In conclusion, activation of ERK1/2 may contribute to BV-induced spontaneous pain and both thermal and mechanical hyperalgesia. Activation of p38 in spinal neurons may be important for the generation of BV-induced thermal hyperalgesia and in microglia may be involved in the maintenance of BV-induced thermal hyperalgesia.

## Methods

### Animals and bee venom administration

The experiments were performed on male Sprague-Dawley albino rats weighted 250–300 g. The animals were kept 2–4 per cage under a 12 h/12 h light-dark cycle regime at room temperature (23–24°C), with free access to food and water. All animal experimental procedures were approved by the Hyogo College of Medicine Committee on Animal Research and were performed in accordance with the college's guidelines for the care and use of laboratory animals.

A volume of 50 μl (200 μg) BV solution (crude venom of honey bee, Sigma-Aldrich Inc, St Louis, U.S.A.) dissolved in 0.9% sterile saline was used. Subcutaneous injection of BV was administered into the posterior plantar surface of the hindpaw of rats under ether anesthesia as reported previously [2].

### Implantation of intrathecal catheters and administration of inhibitors

For chronic and continuous intrathecal drug administration, rats were implanted with catheters as described previously [36,39,40]. In brief, under anesthesia with sodium pentobarbital (40 mg/kg, i.p.), an L5 vertebrae laminectomy was performed, and a soft tube (Silascon, Kaneka Medix Company, Osaka, Japan; outer diameter, 0.64 mm) was inserted into the subarachnoid space of the spinal cord and advanced 3 cm rostrally to the level of the lumbar enlargement (the L4/L5 level) *via *an incision in the dura. Then the muscle incision was sutured and a small subcutaneous pocket was made by spreading apart the subcutaneous connective tissue behind the incision. Next, an Alzet mini-osmotic pump (Model 1003D, CA, U.S.A. providing 72 h of drug delivery) filled with a p-p38 inhibitor, 4-(4-fluorophenyl)-2-4-methylsulfonyl-phenyl)-5-(4-pyridyl)-1H-imidazole (SB203580, Calbiochem, La Jolla, CA, U.S.A., in 10% dimethylsulphoxide (DMSO)) or a potent and specific MEK inhibitor, 1,4-Diamino-2,3-dicyano-1,4-bis (2-aminophenylthio) butadiene (U0126, 1 μg/μl in 10% DMSO) (Calbiochem, La Jolla, CA, U.S.A.), or vehicle (10% DMSO) was put into the pocket and connected to the tube. The pump was soaked in sterile saline overnight prior to pump implantation. The rats were housed individually after surgery and only those without motor disturbance and other neurological deficits were included for further experiments. Two doses of SB203580 (0.5, 2.5 μg/μl) dissolved in 10% DMSO were used. The doses of these inhibitors were determined on the basis of our preliminary experiments [41,42].

### Immunohistochemistry

At appropriate times, control and BV-inflamed rats were deeply anesthetized with sodium pentobarbital (50 mg/kg, i.p.) and then perfused through the ascending aorta with 1% paraformaldehyde in 0.1 M phosphate-buffer (PB, pH 7.4), followed by 4% paraformaldehyde in 0.1 M PB. After perfusion, the L4/L5 spinal cords were removed and postfixed in the same 4% fixative overnight at 4°C and dehydrated by immersion in 20% sucrose in 0.1 M PB at 4°C overnight. The tissue was embedded with Tissue-Tek (Sakura Finetek, Co Ltd, U.S.A.) and frozen in dry ice powder. Transverse sections were cut into 16 μm thick sections at -28 °C in a cryostat.

The sections were processed for immunohistochemistry using the ABC method according to the floating procedure. Sections were blocked with 10% normal goat serum in 0.1 M PBS (pH 7.4) for 1 hr at RT and incubated with one of the following primary antibodies: anti-p-p38 antibody (rabbit anti phospho-p38, Thr180/Tyr 182; 1:400; Cell Signaling Technology, U.S.A.) or anti-p-ERK1/2 (rabbit anti phospho-p44/42 MAP kinase; 1:1000; Cell Signaling Technology, U.S.A.), over two nights at 4°C. The sections were then incubated overnight at 4°C with biotinylated secondary antibody (goat anti rabbit, 1:400; Vector, Germany).

For double immunofluorescence, sections were incubated with a mixture of rabbit anti-p-p38/p-ERK1/2 antiserum and mouse monoclonal anti-neuronal specific nuclear protein (NeuN) (neuronal marker, 1:2000; Chemicon, Temecula, CA) or mouse monoclonal anti-glial fibrillary acidic protein (GFAP) antiserum (astrocyte marker, 1:2000; Chemicon, MA, U.S.A.) over two nights at 4°C, followed by a mixture of Alexa Fluor 488 or Alexa Fluor 594 fluorescence conjugated secondary antibodies (1: 1000; Molecular Probes, OR, U.S.A.) overnight at 4°C. The double-stained images were examined with an Axiovert/LSM510 confocal scanning microscope (Carl Zeiss Microimaging, Inc., Germany). The tyramide signal amplification (TSA Indirect Method, Product NEL 700A, PerkinElmer Life Sciences, Boston, MA) fluorescence procedures [36] were used for double immunofluorescent staining p-p38/p-ERK1/2 with ionized calcium binding adaptor molecule 1 (Iba1, microglia marker, 1:1000; Wako, Tokyo, Japan).

### Behavioral test

We detected both thermal and mechanical hyperalgesia in the injected paw before or at 1 hr, 2 hr, 3 hr, 5 hr, 8 hr, 1 d, 2 d, 3 d and 5 d after BV injection. Mechanical hyperalgesia was assessed with an automated von Frey-type system [43,44], the dynamic plantar aesthesiometer (Ugo Basile, Comerio, Italy). To measure rat hindpaw mechanical thresholds, rats were placed in plastic cages with a wire mesh floor and allowed to acclimate for 2 hr before each test session. A paw-flick response was elicited by applying an increasing force (measured in grams) using a plastic filament (0.5 mm diameter) focused on the plantar surface of the ipsilateral hindpaw. The force applied was initially below detection threshold and then gradually increased from 1 to 50 g over 20 s, then held at 50 g for a further 10 s. The rate of force increase was 2.5 g/s. The force applied to elicit a reflex removal of the ipsilateral hindpaw was considered to be the threshold of mechanical pain. At least three measurements at 5 min intervals were taken at each time-point and the mean of three measurements was considered the paw withdrawal threshold.

To examine thermal hyperalgesia, the rats were placed in a plastic chamber on the surface of a 2 mm thick glass sheet and a radiant heat stimulus from the Plantar Test (7370 Ugo Basile, Italy) was applied to the injection site of the hindpaw. The heat stimulus was terminated with a withdrawal response, or at 20 s to avoid skin damage. The paw withdrawal latency was defined as the duration from the beginning of heat stimuli to the occurrence of the hindpaw withdrawal reflex. Three stimuli were repeated for each site and paw withdrawal thermal latency was obtained by obtaining the mean. The inter-stimulus interval for each heat test at the same region was 5 min.

### Quantitative and statistical analysis

To obtain the number of the immunoreactive cells in the spinal cord sections, 5 sections were randomly selected from each rat, and the mean number from these 5 sections was considered the number of immunoreactive cells per section/rat. The number of immunoreactive cells in the spinal dorsal horn was counted from laminae I-II of spinal cord.

Data were expressed as mean ± S.E.M. Differences in changes of values over time of each group were tested using T-tests and one-way or two-way repeated ANOVA, followed by individual *post-hoc *comparisons (Turkey test). A difference was accepted as significant if p < 0.05.

## Abbreviations

ANOVA: Analysis of variance; BV: Bee venom; CFA: Complete Freund's adjuvant; DMSO: Dimethyl sulfoxide; DRG: Dorsal root ganglia; ERK: Extracellular signal-regulated protein kinase; IR: Immunoreactivity; JNK: c-Jun N-terminal kinase; MAPK: Mitogen activated protein kinase; MEK: MAPK/ERK kinase.

## Competing interests

The authors declare that they have no competing interests.

## Authors' contributions

XYC, YD: Major data collection; data analysis, paper writing.

SLW: Morphological data collection.

HY, KO, KK: Data interpretation.

YD, JC, KN: Project conception and design, paper writing.
